# Vulnerability of avian populations to renewable energy production

**DOI:** 10.1098/rsos.211558

**Published:** 2022-03-30

**Authors:** Tara J. Conkling, Hannah B. Vander Zanden, Taber D. Allison, Jay E. Diffendorfer, Thomas V. Dietsch, Adam E. Duerr, Amy L. Fesnock, Rebecca R. Hernandez, Scott R. Loss, David M. Nelson, Peter M. Sanzenbacher, Julie L. Yee, Todd E. Katzner

**Affiliations:** ^1^ U.S. Geological Survey, Forest and Rangeland Ecosystem Science Center, Boise, ID 87648, USA; ^2^ Department of Biology, University of Florida, Gainesville, FL 32611, USA; ^3^ Renewable Energy Wildlife Institute, Washington, DC 20006, USA; ^4^ U.S. Geological Survey, Geosciences and Environmental Change Science Center, Denver Federal Center, Denver, CO 80225, USA; ^5^ U.S. Fish and Wildlife Service, Carlsbad Fish and Wildlife Office, Carlsbad, CA 92008, USA; ^6^ Bloom Research Inc., Santa Ana, CA 92705, USA; ^7^ Desert District Office, U.S. Bureau of Land Management, Palm Springs, CA 92262, USA; ^8^ Department of Land, Air and Water Resources, University of California, Davis, CA 95616, USA; ^9^ Wild Energy Initiative, John Muir Institute of the Environment, University of California, Davis, CA 95616, USA; ^10^ Department of Natural Resource Ecology and Management, Oklahoma State University, Stillwater, OK 74078, USA; ^11^ Appalachian Laboratory, University of Maryland Center for Environmental Science, Frostburg, MD 21532, USA; ^12^ U.S. Fish and Wildlife Service, Palm Springs Fish and Wildlife Office, Palm Springs, CA 92262, USA; ^13^ U.S. Geological Survey, Western Ecological Research Center, Santa Cruz, CA 95060, USA

**Keywords:** solar, wind, bird populations, wildlife mortality, anthropogenic effects

## Abstract

Renewable energy production can kill individual birds, but little is known about how it affects avian populations. We assessed the vulnerability of populations for 23 priority bird species killed at wind and solar facilities in California, USA. Bayesian hierarchical models suggested that 48% of these species were vulnerable to population-level effects from added fatalities caused by renewables and other sources. Effects of renewables extended far beyond the location of energy production to impact bird populations in distant regions across continental migration networks. Populations of species associated with grasslands where turbines were located were most vulnerable to wind. Populations of nocturnal migrant species were most vulnerable to solar, despite not typically being associated with deserts where the solar facilities we evaluated were located. Our findings indicate that addressing declines of North American bird populations requires consideration of the effects of renewables and other anthropogenic threats on both nearby and distant populations of vulnerable species.

## 1. Introduction

Expanding global demand for energy and the impacts of climate change on human and natural systems have fostered rapid and recent worldwide development of renewable energy. For example, although commercial wind energy generation has occurred for nearly 40 years in the United States, capacity has increased nearly 300% since 2009. The current installed capacity is now greater than 107 gigawatts (GW) from approximately 59 000 turbines [1–3], with a projected capacity greater than 160 GW by 2030 [4]. Likewise, the capacity of utility-scale solar energy, including photovoltaic (PV) and concentrating solar power (CSP) technologies, has increased 9400% in the United States, from 0.4 GW in 2009 to greater than 38 GW in 2019, and is anticipated to exceed 75 GW within 5 years [5]. Worldwide, wind energy capacity (540 GW in 2017) and PV technologies (438 GW in 2017) are forecast to increase by greater than 60 GW yr^−1^ and greater than 80 GW yr^−1^, respectively, through 2025 [6,7].

This growth raises concerns about the potential impacts of renewable energy on the environment when shifting energy production from fossil fuels to reduce the effects of anthropogenic climate change on wildlife species [8–11]. For example, substantial numbers of birds and bats are found dead at some wind and solar energy projects (e.g. wind turbines of many types and sizes, PV panels, CSP parabolic troughs and CSP power towers). Fatalities of birds predominantly are thought to be caused by collisions with turbine blades, PV panels and heliostat solar reflectors, but birds also are killed by concentrated beams of sunlight at CSP power towers, unintentional grounding at solar facilities and drowning in wastewater evaporation ponds at CSP facilities [12–15].

Despite concerns about the sometimes large numbers of wildlife fatalities caused by renewable energy, population-level effects of fatalities are essentially unknown for nearly all species [16–20], with a few exceptions [21–23]. Within the United States, the best estimate is that 140 000–328 000 bird fatalities occur annually at modern monopole turbines, but this number is derived from data collated approximately 10 years ago and at 57% of current installed capacity [13]. Similarly, solar energy generation at 37% of current capacity was estimated to cause 37 800–138 600 bird deaths per year in the USA, with most of these fatalities in California [12,15,24]. However, these large-scale estimates do not account for the effects of renewable energy on populations of individual species, information that is crucial to taxon-based conservation efforts [19,25] and to understanding the vulnerability of community and ecosystem processes affected by birds [26]. Another weakness of these estimates is that they do not distinguish between impacts on locally breeding populations versus impacts that manifest on distant (hereafter ‘non-local’) populations of non-breeding birds that encounter renewable energy facilities on migration or when dispersing.

**Figure 1.**
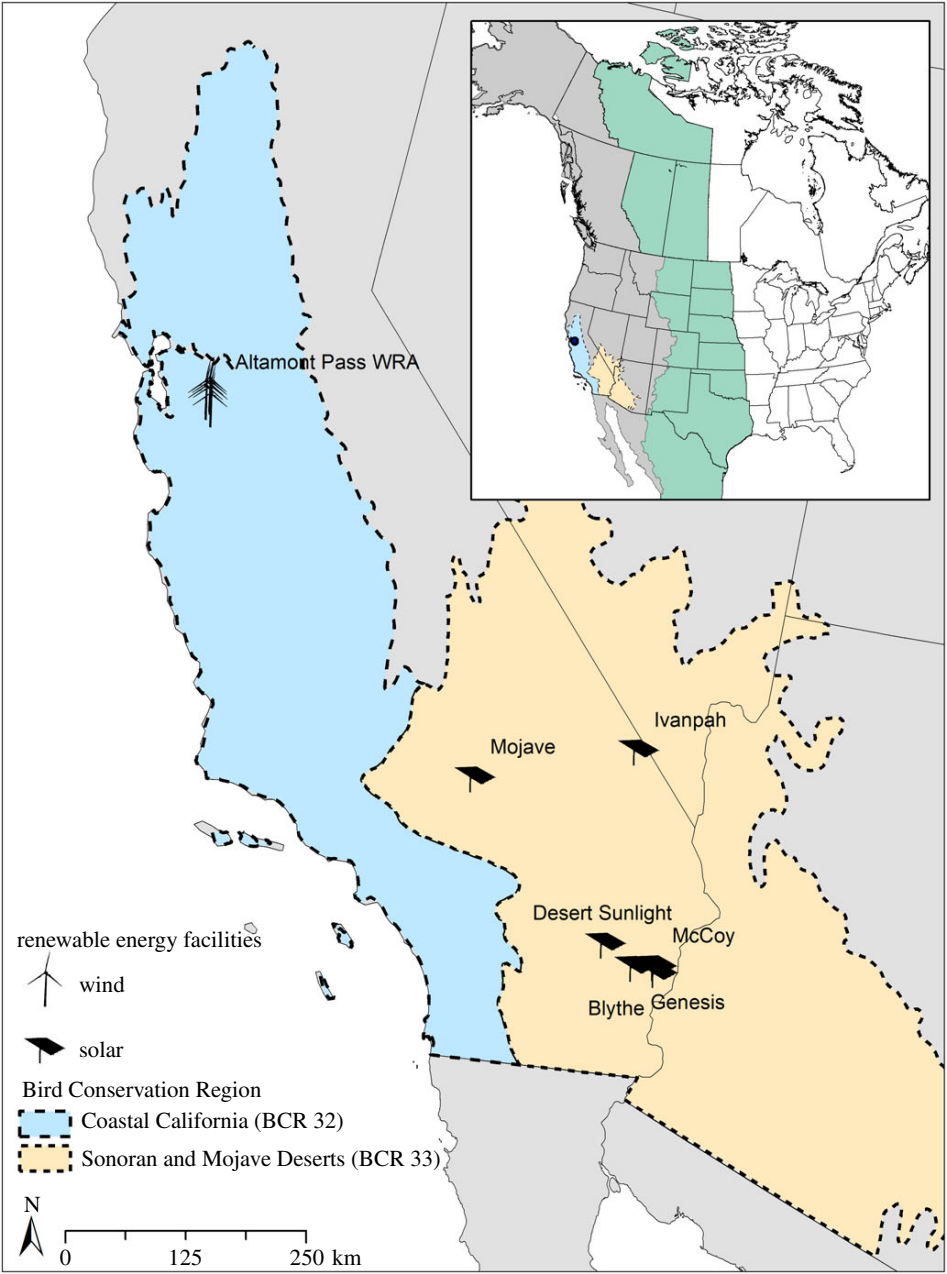
Map of renewable energy facilities in California, USA at which research was conducted. Inset shows the two major western migration flyways of North America (Pacific Flyway in grey; central Flyway in green) that were used with geolocation from stable hydrogen isotope data to define catchment areas (areas holding the sub populations of origin for birds found dead at renewable facilities) to interpret population-level effects. Black solid lines are borders of states and provinces, and dashed lines are borders of Bird Conservation Regions (Coastal California (BCR 32) in blue; Sonoran and Mojave Deserts (BCR 33) in yellow). The Altamont Pass Wind Resource Area (WRA) is composed of approximately 5–30 individual wind facilities located in rolling hills primarily covered with grasslands. See electronic supplementary materials, Methods, for details on both types of facilities.

**Figure 2.**
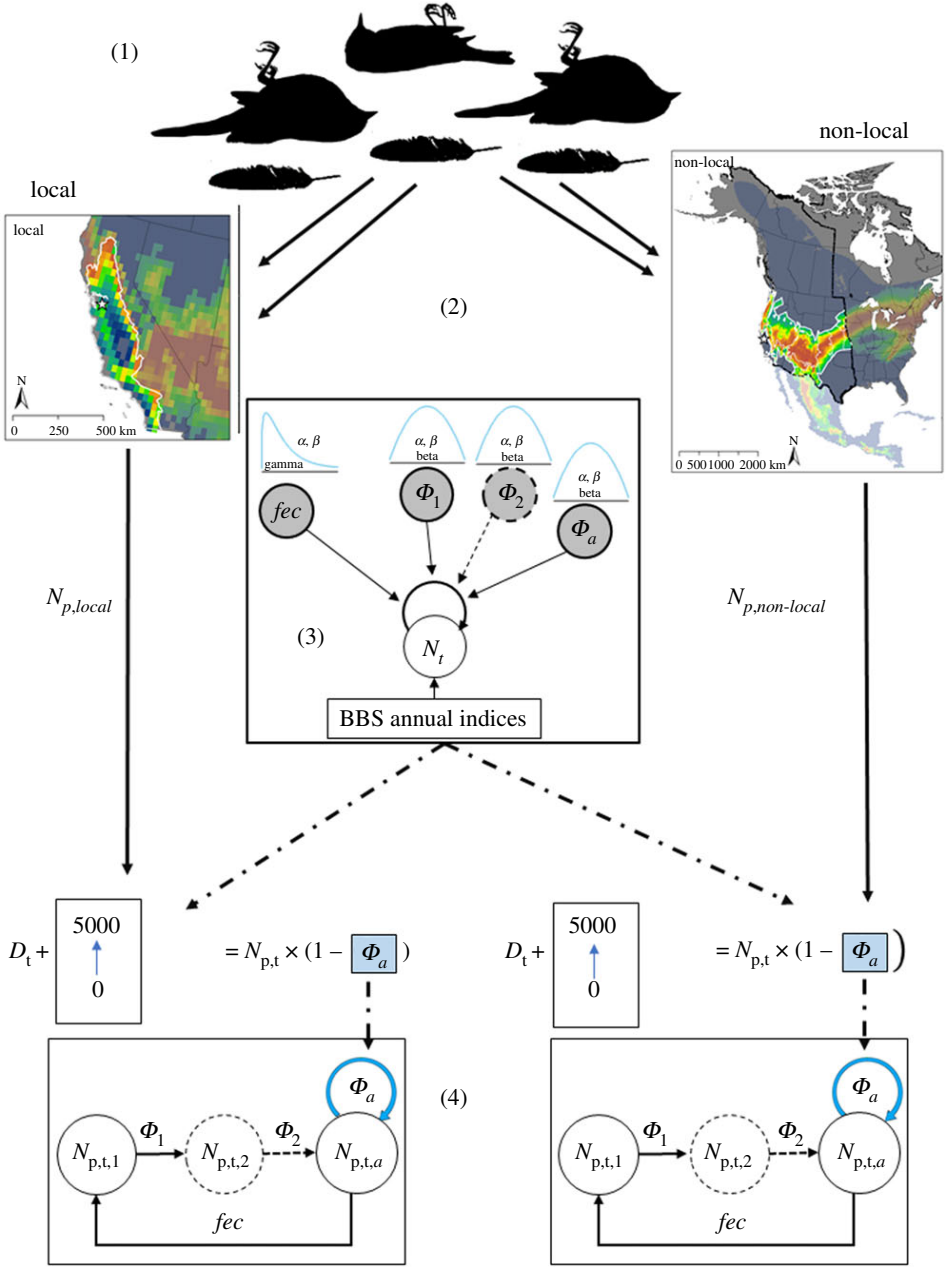
We estimated vulnerability for each priority species by following a framework that included five main steps: (1) determining the geographical origin of fatalities at renewable energy facilities by performing stable hydrogen isotope analysis on avian tissue, (2) defining, for each species, the geographical ‘catchment area’ encompassing the fatalities by using an odds ratio analysis and estimating the size of subpopulations within those catchment areas from existing population estimates, (3) building Bayesian species-specific demographic models to estimate rates of survival, fecundity and population growth, (4) assessing species- and subpopulation-specific vulnerability with sensitivity analyses and a counterfactual ratio (CIU) to estimate the demographic effects of additional fatalities and (5) evaluating taxonomic and ecological correlates of vulnerability (not pictured). Steps 1–4 are illustrated above. For step 3, the rectangle represents the data, circles represent the parameters, and the top of the diagram illustrates the informed prior distributions derived from existing literature for the fecundity (*fec*), and juvenile, immature and adult survival (i.e. *Φ*_1_, *Φ*_2_, *Φ*_a_) parameters, respectively (all shaded in grey). Circles with dashed lines indicate optional parameters for subadult survival, which was only used for some species (see electronic supplementary material, table S5 for model specifications). For step 4, sensitivity analysis of the subpopulation of the catchment area considered absolute changes in annual fatalities (*D*_t_) and the resulting updated *Φ*_a_, (highlighted in blue) then calculated new origin-specific population growth rates (*λ*_s_) under a range of different fatality scenarios (0–5000 additional deaths) while holding constant other demographic parameters. We then used CIU to assess the significance of change from the original *λ* under current conditions to the new *λ*_s_ to estimate vulnerability of subpopulations.

Given the loss of approximately 3 billion birds in North America since 1970 [27] and the ecological, economic and socio-cultural relevance of avian species [28], a major scientific priority is to understand and mitigate the many threats to bird populations. With the anticipated build-out of renewable energy facilities to meet state and federal emission reduction goals [29], a critical component of this priority is to understand species' vulnerability to cumulative incidental deaths from renewable energy development. We applied a comprehensive analytical framework combining geolocation via stable isotope analyses of bird tissue, current population trend data, literature-based survival and fecundity rates, and Bayesian hierarchical population models to evaluate the vulnerability to additional fatalities of a taxonomically diverse suite of 23 priority bird species killed at renewable energy facilities. These species were selected based on stakeholder input using factors such as ecological value, conservation status, and frequency and risk of mortality at wind and solar energy generation facilities in California, United States. We focused on California because it is a global biodiversity hotspot [30] and one of the world's initial locations for wind and solar energy development and innovation (figures 1 and 2 and electronic supplementary material, tables S1 and S2; see Methods for detailed description of species and renewable energy site selection). Our unique approach estimates the number of individuals present in both local and non-local regions (hereafter, local and non-local ‘catchment areas’) as a context against which we evaluate absolute vulnerability and relative risk to additional fatalities for each species, as well as taxonomic and ecological patterns of vulnerability. This approach identifies the extent to which bird species are more or less likely to experience declines of a specified magnitude (i.e. their vulnerability), given the cumulative and range-wide mortality effects of many renewable projects together with other human-caused mortality sources.

## 2. Methods

We used expert opinion and ecological and conservation-related traits to identify a taxonomically diverse suite of 32 priority bird species for study (electronic supplementary material, table S1; also see [31]). We then estimated vulnerability for each using a five-step framework: (1) determining the geographical origin of fatalities at renewable energy facilities, (2) defining the geographical ‘catchment area’ encompassing the fatalities and estimating the size of subpopulations within those catchment areas, (3) building species-specific demographic models to estimate rates of survival, fecundity and population growth, (4) assessing species- and subpopulation-specific vulnerability with sensitivity analyses to estimate the demographic effects of additional fatalities, and (5) evaluating taxonomic and ecological correlates of vulnerability (figure 2). Steps 1–4 of this process are defined in Katzner *et al*. [18], implemented here with context-specific alterations and improvements (see electronic supplementary material for full details on the methods).

### 2.1. Geographical origin of individuals killed at renewable energy facilities

We obtained feathers from avian fatalities at renewable energy facilities in California, including from approximately 5–30 wind facilities at the Altamont Pass Wind Resource Area (APWRA) in Alameda and Contra Costa Counties [32] and six solar facilities in Riverside and San Bernardino Counties (figure 1). The wind and solar facilities in these two areas represent a wide variety of technology and production types (e.g. wind turbines of multiple models, manufacturers and power generation capacities, as described in greater detail in the electronic supplementary material). We measured stable hydrogen isotope (*δ*^2^H) values in feather samples, together with information on moult location and scaled *δ*^2^H values of precipitation at the site of feather growth [33–35], to assign a local or non-local origin for each bird (i.e. its ‘subpopulation of origin’). This assignment was based on whether the collection site (renewable facility) was located within the area of the species’ range that exceeded a 5 : 1 odds ratio (OR) threshold value [17].

### 2.2. Define the geographical catchment area for the population of interest

We used information from species range maps, migration flyways in the western US, Bird Conservation Regions (hereafter ‘BCRs’) [36] and local and non-local subpopulation of origin assignments to delineate species- and subpopulation-specific ‘catchment areas’ [17,37] (electronic supplementary material, methods and figure S1). Each local catchment area included the aggregate mean summary surface greater than or equal to 5 : 1 OR within the local BCR that contained the renewable energy facility of interest (i.e. wind facilities were in the Coastal California BCR (BCR 32) and solar facilities in the Sonoran and Mojave Desert BCR (BCR 33)). Non-local catchment areas included the aggregate mean summary surface greater than or equal to 5 : 1 OR for all non-local complete or partial BCRs within the Central and Pacific flyways [38]. We excluded all full or partial BCRs within Mexico, as that country lacked species-specific population estimates.

After defining local and non-local catchment area boundaries for each species, we estimated the total number of individuals in each catchment area population (hereafter ‘*N*_p_’) using BCR-specific mean population estimates from Partners in Flight (PIF) databases [39,40]. We generated species-specific estimates of population size by weighting estimates by the stressor-affected proportion of the catchment area (i.e. the sum of the OR values across pixels within the greater than or equal to 5 : 1 OR aggregate mean summary surface catchment area divided by total pixels in that area). For BCRs extending beyond the Central and Pacific flyways, we similarly weighted population estimates to only include the proportion of the BCR located within the catchment area. Finally, we estimated total $N_{p}$ for each subpopulation *s,* using the adjusted population estimate for the local BCR (BCR 32 or BCR 33) to estimate the number of birds within the local catchment area, or summing the adjusted population estimates among all BCRs (excluding the local BCR) within the non-local catchment area for the non-local $N_{p}$ (electronic supplementary material, figure S1) [39]. We also accounted for uncertainty in population size estimates by generating $N_{p}$ values based on lower and upper 95% bounds (i.e. 95% LCI and 95% UCI) of BCR-specific population estimates from the PIF databases [39,41].

For species lacking BCR-specific population estimates, we used continental-scale (USA and Canada) PIF estimates $\left(C_{p}\right)$, scaling population estimates by the proportion of the range within the defined catchment area. For species with no available isotope data, we defined a non-local catchment area from the portion of the species range [42] within the Central and Pacific flyways in the USA and Canada (excluding the local BCR) and weighted the range-wide population estimate accordingly. In both of these cases, we assumed equal distribution of individuals across the species’ range. To generate hypothetical 95% LCI and 95% UCI for these populations for subsequent analyses, we assumed lower and upper population size limits to be one half and double the continental-level population estimates, respectively (i.e. 95% LCI = 0.5 × *C*_p_; 95% UCI = 2 × *C*_p_). Lower and upper 95% limits for focal species with BCR-specific estimates ranged from 0.52 to 1.63 × *C*_p_, respectively, which suggests that our hypothetical limits of 0.5 to 2.0 × *C*_p_ were sufficiently broad to represent uncertainty in population estimates for species with only continental-scale values.

### 2.3. Demographic models

For each priority bird species, we used a Markov chain Monte Carlo (MCMC) approach, within a Bayesian hierarchical modelling framework to generate multi-age matrix population models and estimate species-specific demography parameters (i.e. survival and fecundity) and annual population growth rates (hereafter ‘*λ*’) [43–46] (figure 2; electronic supplementary material, table S3). The modelling was implemented with JAGS v. 4.3.0 and R package *jagsUI* [47,48]. We used literature-derived mean and SE values of survival and fecundity to generate prior distributions (electronic supplementary material, table S4) and used North American Breeding Bird Survey (BBS) species-specific annual indices of mean individuals detected per survey route in California from 1968 to 2015 [49]. When California-specific indices were unavailable, we substituted annual indices for western North America. Similarly, if species-specific data were unavailable in the literature to generate informative prior distributions for survival and fecundity, we used mean and SE values from confamilials with the best available demographic data (electronic supplementary material, table S4).

Model structure and complexity varied by species due to both availability of demographic data in the literature to inform model priors, and due to species-specific differences in demographic parameters (i.e. number of life stages, age of first breeding). Our least complex model incorporated a 2 × 2 matrix model for juvenile and adult stage classes (e.g. horned lark, *Eremophila alpestris*). By contrast, golden eagles (*Aquila chrysaetos*) reach breeding age at 4 + years and thus required a 5 × 5 matrix with three modelled stage classes (i.e. juvenile, immature, adult) (electronic supplementary material, table S5).

For each species, we constructed three candidate models with increasing constraints on model priors for mean survival ‘ϕ‘ ($\mu_{\phi };$ all age classes) and mean fecundity $\left(\mu_{f}\right)$ to limit non-identifiability of model parameters, including: (i) no additional constraints on prior distributions, (ii) additional constraints on survival of $\mu_{\phi }\pm 0.2$ , and on fecundity of $\mu_{f}\pm (0.5\times \mu_{f})$, and (iii) additional constraints on survival of $\mu_{\phi }$ ± 0.1, and on fecundity of $\mu_{f}\pm (0.25\times \mu_{f})$. We ran models by sampling from nine independent Markov chains with a burn-in of 50 000, thinning of 1000 and 200 000 subsequent iterations.

To assess model convergence and select the best-fit model for subsequent analysis we compared the deviance information criterion (DIC) values across models, selected models with *R̃* values < 1.1, visually inspected traceplots to assure models converged, and reviewed the biological feasibility of the posterior distributions [50,51]. If initial model runs did not converge, we updated models with an additional 200 000 iterations. After reassessing the updated model fit, if models still failed to converge, we removed that species from subsequent analyses [50] (electronic supplementary material, table S1). See the electronic supplementary material for further details regarding model construction and implementation.

### 2.4. Subpopulation-specific vulnerability

To estimate potential vulnerability of each species to additional deaths beyond those in current conditions, we simulated the sensitivity of each subpopulation to both absolute and proportional increases in numbers of fatalities. We first estimated the current total number of annual fatalities from all sources in year *t* for each local and non-local catchment subpopulation *s* $\left(D_{s,t}\right)$ by multiplying catchment area subpopulation size $\left(N_{p_{s,t}}\right)$ by a mortality rate of 1 – adult survival $\left(\Phi_{a}\right)$:

2.1 $$D_{s,t}=N_{p_{s,t}}\times (1-\Phi_{a}).$$

To evaluate sensitivity of the catchment area subpopulation to absolute changes in numbers of fatalities, we calculated new estimates of growth rates for each local and non-local population, $\lambda_{s},$ under a range of different fatality scenarios (adding 100 to 5000 to the estimated current adult fatalities if $N_{p_{s,t}}\geq 5000$, otherwise adding 100 to $N_{p_{s,t}}$ fatalities) while holding constant other demographic parameters (i.e. juvenile and immature survival and fecundity rates) (figure 2; electronic supplementary material, table S3). We first increased $D_{t,s}$ in increments of 100 additional fatalities and ran 1000 iterations of a 48-year time series (i.e. the same duration as that of the BBS dataset) using the revised mean adult survival $\left(\mu_{\phi }\right)$ to generate a distribution of the new growth rate for each population, $\lambda_{s}$.

We assessed the significance of change from the original λ under current conditions to new $\lambda_{s}$ using a counterfactual ratio of impacted subpopulations to the original population ($\lambda_{s}/\lambda_{\mathrm{original}}$, hereafter ‘CIU’; electronic supplementary material, table S3) [18,52–54]. We classified species as ‘moderately vulnerable’ if vulnerability (1 – CIU) ≥ 0.2 (i.e. greater than or equal to 20% reduction in λ) after less than or equal to 5000 additional fatalities and ‘highly vulnerable’ if 1 – CIU ≥ 0.2 after less than or equal to 1000 additional fatalities. To calculate the sensitivity of the subpopulation of the catchment area to *proportional* changes in fatalities, we repeated this sensitivity analysis, increasing fatalities at intervals of 1% from 1–50% of the size of the catchment area subpopulation $N_{p_{s}}$. The 1000 and 5000 fatalities used in the thresholds have a high degree of support for the species we modelled, given the potential for fatalities from renewable energy under current operating capacity in local BCRs [20,55] and estimates of *absolute* numbers of additional fatalities cumulatively expected under different future build-out scenarios [20,55,56]. Estimates of mortality at current levels of build-out suggest that many species are probably experiencing greater than or equal to 1000 fatalities per year in North America due to renewable energy [57]. For example, Altamont Pass WRA alone is estimated to kill 150–200 red-tailed hawks and 100–500 horned larks per year [32,58]. Given the anticipated build-out of renewable facilities at 5–10 × current levels in the coming decades [3,5], 1000–5000 annual fatalities within a catchment area represents a realistic range of potential bird mortality.

We also calculated relative risk (*RR*_s_) to subpopulations to determine if the incidence of local fatalities in the isotope samples as a proportion of the local subpopulation (i.e. local fatality rate; *F*_l_), was equal to the incidence of non-local fatalities in samples as a proportion of the non-local population (non-local fatality rate; *F*_n_):

2.2 $$RR_{s}=\frac{F_{l}}{F_{n}}\;\;=\frac{\mathrm{no}.\;\mathrm{local}\;\mathrm{fatalities}/\mathrm{local}\;\mathrm{population}\;\mathrm{size}}{\mathrm{no}.\;\mathrm{non}\text{-}\mathrm{local}\;\mathrm{fatalties}/\mathrm{non}\text{-}\mathrm{local}\;\mathrm{population}\;\mathrm{size}}\;.$$

An estimated ratio greater than 1.0 suggested increased risk for birds in the local subpopulation (i.e. more local individuals killed than expected) and a ratio less than 1.0 indicated increased risk for birds in the non-local subpopulation (i.e. more non-local individuals killed than expected). For more details on sensitivity analyses, see the electronic supplementary material.

### 2.5. Taxonomic and ecological correlates of vulnerability

To understand factors potentially leading to variation in vulnerability between and within each energy type (solar and wind) and category of geographical origin (local and non-local), we evaluated differences of vulnerability, as defined above, among taxonomic guilds, migration strategies, habitat types, and sizes of the population or subpopulation. Taxonomic correlates we considered were avian order and species groups that commonly appear in monitoring reports from wind and solar facilities (waterbirds, raptors, passerines, other) (electronic supplementary material, table S1). Migratory strategies we considered were based on time of day of migration (e.g. diurnal, nocturnal) and migratory strategies (e.g. resident, migrant, partial migrant) [59]. Habitat-related correlates we considered were based on the primary habitat type used by each species [60]. To evaluate correlates of population size, we used estimates of the species- and origin-specific catchment area (*N*_p_) and continental (USA and Canada) population [39,40] as outlined above.

## 3. Results

**Figure 3.**
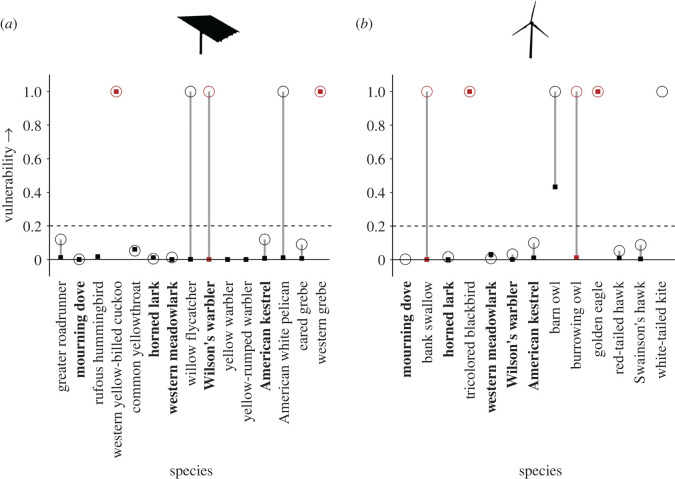
Vulnerability (ranges from 0 (low) to 1 (high), as defined in Methods) after increases in simulated deaths for local (○) and non-local (▪) populations of 23 priority bird species found dead at (*a*) solar and (*b*) wind energy facilities in California, USA. Species in black were classified as moderately vulnerable (vulnerability greater than 0.2 after simulated fatality of 5000 adult individuals). Those highlighted in red were classified as highly vulnerable (vulnerability greater than 0.2 after 1000 additional deaths) based on effects on populations in local or non-local catchment areas. Vertical lines connect local and non-local populations. The five species with names in bold were found dead at both types of energy facility.

Our results highlight, for the first time, distinct patterns of population- and subpopulation-level vulnerability for a wide variety of bird species found dead at renewable energy facilities. Of the 23 priority bird species killed at renewable facilities, 11 (48%) were either highly or moderately vulnerable, experiencing a greater than or equal to 20% decline in the population growth rates with the addition of up to either 1000 or 5000 fatalities, respectively (see Methods for detailed derivation of vulnerability). For five of these 11 species, killed birds originated both locally and non-locally, yet vulnerability occurred only to the local subpopulation (table 1, electronic supplementary material, tables S5–S7; figure 3, electronic supplementary material, figure S4). Casualties of one additional species (white-tailed kite, *Elanus leucurus*) originated from only a local population, which was also vulnerable. For the other five species, dead birds originated from both local and non-local subpopulations and vulnerability also occurred to both. These five species included western yellow-billed cuckoo (*Coccyzus americanus occidentalis)* and western grebe (*Aechmophorus occidentalis)* killed at solar facilities and tricolored blackbird (*Agelaius tricolor*), barn owl (*Tyto alba*) and golden eagle (*Aquila chrysaetos*) killed at wind facilities. Beyond vulnerability, relative risk (i.e. based on the comparison between local and non-local fatality rates within a species, as defined in Methods) was disproportionately high for local subpopulations of horned lark, Wilson's warbler (*Cardellina pusilla*) and burrowing owl (*Athene cunicularia*) affected by wind facilities; local subpopulations of western meadowlark (*Sturnella neglecta*), Wilson's warbler and greater roadrunner (*Geococcyx californianus*) affected by solar facilities (table 1); and non-local subpopulations of western meadowlark and American kestrel (*Falco sparverius*) affected by wind facilities.

**Table 1.** Vulnerability (ranges from 0 (low) to 1 (high), as defined in Methods) and relative risk for 23 priority bird species found dead at renewable energy facilities in California, USA. Shown are results of sensitivity analysis evaluating absolute population vulnerability to simulation of an additional 1000 (1 k) and 5000 (5 k) deaths, and relative risk for local and non-local subpopulations (<1 is less risk than expected, >1 is more risk than expected; 95% confidence intervals for relative risk presented in parentheses). Light grey indicates species vulnerable to an additional 5000 adult deaths; dark grey indicates species vulnerable to 1000 additional adult deaths. Dashes indicate no analysis presently possible for that species and energy type.

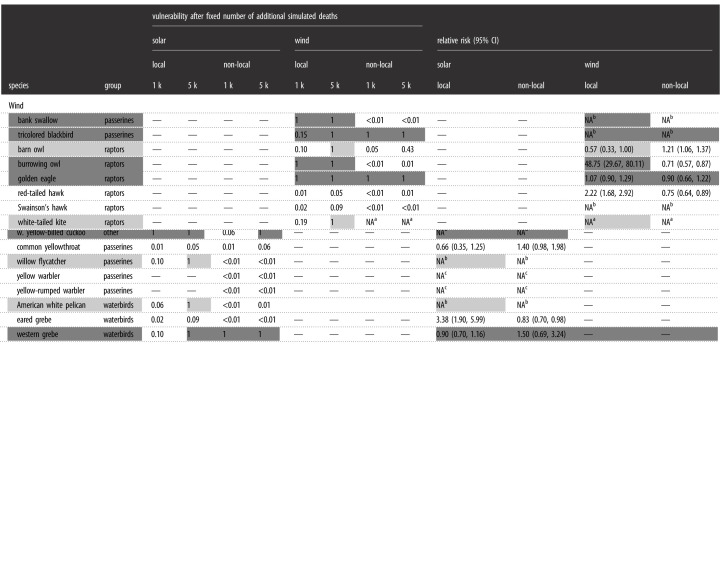

^a^Unable to calculate vulnerability for non-local population or relative risk; all samples classified as local individuals.

^b^No available carcasses for stable isotope analysis.

^c^Unable to calculate relative risk; greater than 98% of all samples classified as non-local individuals.

Vulnerability varied by species and by taxonomic group. Highly vulnerable species included those with already small, declining or range-restricted populations (tricolored blackbird, western yellow-billed cuckoo) that were affected by additional fatalities numbering as few as 1% of their populations (electronic supplementary material, figure S6). However, vulnerable species also included those with larger, stable populations or more widespread ranges, including a waterbird (western grebe), raptors (golden eagle, burrowing owl) and songbirds (Wilson's warbler, bank swallow (*Riparia riparia*)) (figure 3, electronic supplementary material, figures S7–S9). Raptors showed greater among-species variability to proportional increases in numbers of fatalities than any other taxonomic group, although most raptors also were vulnerable to increases in absolute numbers of fatalities (electronic supplementary material, figure S6).

**Figure 4.**
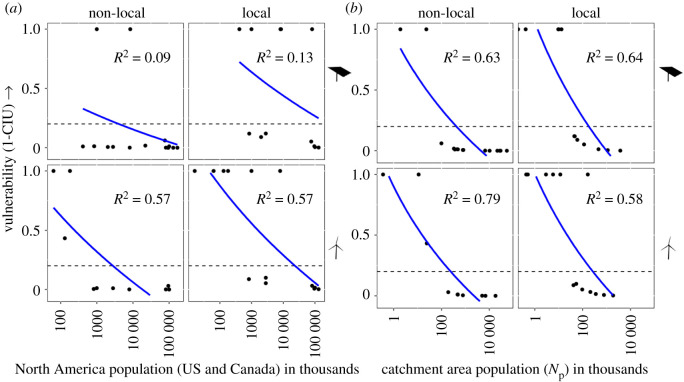
Vulnerability (ranges from 0 (low) to 1 (high), as defined in Methods) after increases in simulated additional deaths of 5000 adult individuals for populations of 23 priority bird species found dead at solar and wind energy facilities in California, USA. Vulnerability is shown for non-local and local portions of populations, by energy type (solar or wind on top and bottom panels, respectively), and as a function of number of individuals in (*a*) the North American population and (*b*) the catchment area population. Horizontal dashed line indicates vulnerability of 0.2; blue line and *R*^2^ value represents smoothed regression line where *y* = log_10_(*x*).

Patterns in vulnerability were driven more by the size of the subpopulation in the specific local or non-local catchment area affected by renewables (figure 4*b*, electronic supplementary material, figure S5b,d), than by the size of the continental, range-wide population (figure 4*a*, electronic supplementary material, figure S5a,c). Regardless of continental population size, the impacted subpopulations for most vulnerable species spanned a narrow geographical range or had low abundance within the catchment area. For example, willow flycatchers (*Epidonax traillii*) have a continental population of greater than 8 million birds, but the vulnerable local catchment area subpopulation near the solar facilities included in our dataset is less than 10 000 individuals. Thus, the local subpopulation of this species is highly vulnerable to effects from increased fatalities at renewable energy facilities in the Mojave Desert (table 1, electronic supplementary material, table S7).

Despite the substantial ecological, morphological, behavioural, and demographic differences among taxonomic guilds (e.g. raptors, waterbirds) represented in the priority species we considered, we observed few distinct taxonomic patterns in vulnerability across these groups. By contrast, there were patterns in vulnerability associated with habitat use, migratory strategy, migratory timing and energy type (electronic supplementary material, figures S7–S9). For example, all but one species identified as vulnerable to mortality at wind energy facilities had both year-round resident subpopulations that include California and diurnally migratory subpopulations that pass through or winter in the state. Species identified as vulnerable to solar were predominantly nocturnal migrants, including several passerines and a waterbird, with both resident and migratory subpopulations (electronic supplementary material, figures S8 and S9). However, of five species vulnerable to solar, most (60%) were vulnerable only in the local catchment area. Again, the exceptions to this were the western yellow-billed cuckoo and western grebe, which had vulnerable subpopulations in both local and non-local catchment areas.

Vulnerability was associated with local habitat conditions for wind energy, but less so for solar energy. Most focal species vulnerable to mortality from wind energy were grassland- or wetland-associated species, possibly because our wind dataset only included facilities in grassland habitats (figure 1). By contrast, the species vulnerable to mortality at solar facilities were not associated with deserts, the dominant habitat type near the solar facilities in our dataset, but instead included scrub, woodlands and water-associated species (electronic supplementary material, table S1).

## 4. Discussion

This study shows that many of the bird species killed at renewable energy facilities are vulnerable to population or subpopulation-level effects from potential increases in fatalities from these and other anthropogenic mortality sources. About half (48%) of the species we considered were vulnerable, and they spanned a diverse suite of taxonomic groups of conservation concern that are resident to or that pass through California. The inference from these analyses applies to the set of focal species and taxonomic groups analysed here, which represent only a small fraction of all bird species killed by renewable energy facilities. Nevertheless, our models highlight the relevance of understanding species-specific, population-level vulnerability for these and other species. Although birds face many human-caused threats, focusing on species of concern that are found dead at renewable facilities greatly improves understanding of the impacts of these and other anthropogenic developments.

Critically, not only local but also non-local, and often very distant, subpopulations often were vulnerable to additional fatalities at California renewable energy facilities. This matters because nearly all environmental monitoring conducted at renewable energy facilities evaluates local subpopulations (e.g. [12–15]) to infer population-level consequences of fatalities. Our results illustrate that such locally focused surveys may poorly predict the cumulative impacts of fatalities. This study, therefore, emphasizes the importance of assessing the origins of wildlife affected when interpreting consequences to wildlife populations of these, or any, types of anthropogenic activities.

Our vulnerability scores were based on fixed numbers of annual fatalities (1000 and 5000). While this magnitude of mortality is unlikely at any single renewable energy project, it is reasonable when considering cumulative effects of many renewable projects at once [20,55,56], or when considering renewable energy effects combined with other human-caused sources of bird mortality. In fact, this study shows that because renewable energy may affect both local and non-local subpopulations (including distant subpopulations of migratory species [31]), cumulative effects of renewable energy probably are more extensive than previously understood, especially for migratory species. Such non-local demographic effects only rarely have been documented for renewables or for other anthropogenic mortality sources (i.e. [61,62]).

Although our models were relatively sophisticated compared with analyses used in many past evaluations of renewable effects on wildlife, data limitations prevented incorporation of some demographic processes (e.g. immigration, emigration, Allee effects) that can affect population responses to anthropogenic stressors. Further, we assumed all anthropogenic-related fatalities are additive (i.e. not compensated for at the population level by density-dependent or other processes) [63], that fatalities are constant through time and across avian age classes, and that all simulated fatalities were adults (electronic supplementary material, table S3). Future research could evaluate these processes, as well as alternative assumptions, to improve understanding of renewable effects on bird populations. For example, models addressing full annual cycles, accounting for density dependence, or taking a maximum sustained yield or potential biological removal approach could include detailed information on anthropogenic impacts in each part of the life cycle and may help identify cumulative effects of anthropogenic stressors. However, unlike the approach we used, such analyses currently are limited to species with sufficient information to construct these data-intensive models [22,46,64,65]. Additionally, while our model and its associated assumptions may not reflect true population dynamics, our approach allows for an estimate of the upper limit of potential vulnerability because the absence of density dependence removes any demographic compensation for fatalities [22].

Some of these species-specific data issues could be addressed by increasing systematic data collection at renewable facilities. This is especially true given that rigorous fatality estimates are lacking for most species [66]. However, there is growing interest in many management and permitting efforts to reduce the number of surveys conducted at facilities. As such, future approaches similar to ours may become increasingly reliant on limited datasets to assess species fatality risk and vulnerability. Adapting future models to work with limited datasets while still incorporating metrics such as density dependence, covariance in vital rates, temporal variation in simulated fatalities across multiple age classes, or other factors known to occur in populations affected by anthropogenic fatalities may be beneficial to ongoing efforts to assess species-specific vulnerability.

The susceptibility to fatalities at wind energy facilities of grassland-associated species, especially raptors, may be due to a suite of factors, including the prevailing habitat type at the wind facilities studied, the prolonged period of exposure of the resident birds affected, catchment area population size, and the behaviour and demography of those species present. Many vulnerable grassland raptors, including diurnally active golden eagles and white-tailed kites, and the less diurnal burrowing and barn owls, are year-round residents at the wind energy facilities we studied [32]. All these species also perform flight displays or courtship behaviours to attract mates, search for prey, and defend territories (e.g. [67,68]). Similar diurnal aerial flight is also typical for many other open-country birds (e.g. red-tailed hawk (*Buteo jamaicensis*), horned lark and the vulnerable, aerial-foraging bank swallow [59]). In conjunction with year-round residency and abundance of catchment area populations, these behaviours may be potential indicators for population vulnerability. This line of thought is supported because, although we did not classify subpopulations of horned larks or red-tailed hawks as ‘vulnerable’ to California wind facilities, these species are among the most frequently reported fatalities at wind facilities in North America [14,20].

The majority of current and ongoing wind energy development in the United States is in the native or converted grassland habitat of the Great Plains and Midwest [1] that overlaps with the breeding, wintering or migration ranges for many grassland birds. This suggests open-country species with small catchment area subpopulations may be vulnerable to wind energy if they experience fatalities. In this same vein, it is important to interpret our vulnerability threshold with the demography of individual species in mind. Raptors and other species with slow intrinsic population growth rates have been shown to be more sensitive to changes in adult survival [69,70]. Similarly, any decrease in population growth would be detrimental for those species already experiencing long-term population declines due to factors such as habitat loss and effects of climate change [27,71].

In contrast to wind facilities, for which most vulnerable species were year-round residents, the most vulnerable avian species at solar facilities were non-local migrants. Once again, this pattern of vulnerability is probably due to many of the same factors as at wind facilities, including facility placement, the size of the catchment area subpopulation, and underlying behavioural and demographic factors. The solar facilities we studied are located in low-elevation areas of the Mojave Desert and are in close proximity to important wintering and breeding grounds for songbirds and waterbirds that use the Pacific Flyway in North America. Although we did not detect vulnerability in desert-specialist species found dead at solar facilities in the region (electronic supplementary material, table S1), facility placement along a constricted portion of this flyway may increase vulnerability of species engaged in long-distance migration. Other characteristics of these facilities may also increase vulnerability of migrant birds. For example, some evidence suggests reflection of polarized light from the sun, moon or artificial sources, off solar panels may attract insects or create a ‘lake effect’ mimicking a large body of water, and thereby attract water-associated species such as loons and grebes [24,72]. Further, desert-specialist species in the Mojave Desert region have experienced substantial population declines over the past century due to climate change [10]. These declines highlight the need to address cumulative effects of facility placement on both breeding and wintering avian species in addition to vulnerable migrant subpopulations [11,73].

Despite the patterns of vulnerability we document for birds found dead at renewable energy facilities, there are additional complexities to this problem. For example, the two energy types we considered are actually composed of multiple subtypes, with at least four generations and sizes of wind turbine technology and three different types of solar energy production (see study site in Methods). Likewise, energy facility infrastructure, including transmission lines, roads or fencing, may also contribute to site-specific fatalities. Thus, each facility embodies unique technological and infrastructural attributes, all of which may modulate the type, magnitude and seasonality of species-specific mortality [66]. As such, it may be beneficial to apply this analytical approach in a setting with fewer subtypes of renewable technologies. Furthermore, it is often difficult to evaluate other factors that influence variability and demographic effects of mortality. For example, field survey data from facilities often are not suited to the statistical analyses required to estimate cumulative fatalities across multiple facilities [66], and sample sizes of dead birds are usually too small to assess variation by season, year or energy subtype. Nevertheless, incorporating variation in seasonal, annual, spatial or subtype-specific numbers of fatalities from additional facilities across North America would help clarify mechanisms, modalities and population-level consequences of fatalities at renewable energy facilities.

Despite being the focus of massive conservation efforts [74–76], bird populations across North America have declined by nearly 3 billion individuals in less than 50 years [27], and similar bird declines are occurring across the world (e.g. [77]). Although we focused on direct mortality, renewable energy also may cause indirect and sub-lethal effects, for example, through displacement of birds and disruption of habitat. Furthermore, wind and solar are part of a suite of anthropogenic stressors that are relevant to avian populations. For example, climate change, habitat loss and degradation, pesticides, killing by domestic cats, and collision with transmission lines, vehicles and buildings [25,78–81] all can directly or indirectly affect bird populations. Although the approach we outline here could be used to interpret threats to species affected by these other stressors, most of them are comparatively well-understood. In contrast, infrastructure associated with renewable energy is an emerging and poorly understood threat to birds. As the build-out of renewable energy continues, the habitat and species-specific patterns in vulnerability we modelled for this small set of species affected in a single region will become broadly relevant to a large suite of species across the planet.

This study illustrates, for the first time and for a large, taxonomically diverse suite of priority species, both the vulnerability of a subset of avian populations to renewable energy development, and the manner in which the demographic influence of renewable energy facilities may extend far beyond the site at which fatalities occur. Our inference is underpinned by novel integration of subpopulation size estimates into demographic models within isotopically determined catchment areas. As such, our analyses highlight the importance of incorporating spatial ecology into assessments of the demographic relevance to avian populations of anthropogenic stressors such as renewable energy. Such assessments also have important conservation implications. In the case of renewable energy, decisions about facility siting, investment and development, as well as management and mitigation actions, will be most effective if they consider both local and non-local impacts to focal species, and if their demographic frame of reference extends to breeding, wintering or stopover habitat far from where the facility is located.

## Data Availability

All data and R Code generated or analyzed during this study are available on a public web page hosted by the U. S. Geological Survey (USGS): https://doi.org/10.5066/P9K15P8Y. The data are provided in electronic supplementary material [82].
